# The emergence of the nicotinamide riboside kinases in the regulation of NAD^+^ metabolism

**DOI:** 10.1530/JME-18-0085

**Published:** 2018-05-30

**Authors:** Rachel S Fletcher, Gareth G Lavery

**Affiliations:** Institute of Metabolism and Systems ResearchUniversity of Birmingham, Birmingham, UK

**Keywords:** NAD^+^, metabolism, nicotinamide riboside, energy

## Abstract

The concept of replenishing or elevating NAD^+^ availability to combat metabolic disease and ageing is an area of intense research. This has led to a need to define the endogenous regulatory pathways and mechanisms cells and tissues utilise to maximise NAD^+^ availability such that strategies to intervene in the clinical setting are able to be fully realised. This review discusses the importance of different salvage pathways involved in metabolising the vitamin B3 class of NAD^+^ precursor molecules, with a particular focus on the recently identified nicotinamide riboside kinase pathway at both a tissue-specific and systemic level.

## NAD^+^ as a redox cofactor and signalling molecule

The vital role of NAD^+^ in redox metabolism was first described in 1906 by Arthur Harden as being a required cofactor for alcohol fermentation (Harden & Young 1906). It has since been well established as a redox coenzyme necessary for many redox reactions (Euler-Chelpin 1966). During these redox events NAD^+^ can be reduced by accepting electrons from donor molecules or, in reverse, NADH can be oxidised to NAD^+^ by donating electrons. This interchange of electrons allows catalysis of reversible transhydrogenase reactions (Warburg & Christian 1936). Notably, NAD^+^ redox reactions are vital in energy production pathways such as glycolysis, oxidative phosphorylation and fatty acid oxidation. Typically NAD^+^ is predominantly found in its oxidised form with NAD/NADH ratios varying from 10/1 to 700/1 and a total cellular concentration thought to be estimated between 0.3–1.0 mM in mammalian cells (Veech* et al*. 1972, Casazza & Veech 1986). Alternatively, NAD^+^ can be phosphorylated to NADP^+^ by NAD kinase and likewise this also exists in its reduced form NADPH. NADP(H) concentrations are estimated at 10% of total NAD(H) levels (Veech *et al*. 1972, Casazza & Veech 1986). NADPH acts as an essential reducing agent aiding many anabolic pathways including nucleic acid and lipid biosynthesis (Berger* et al*. 2004, Tedeschi* et al*. 2016).

More recently, NAD^+^ has been recognised as an important signalling molecule that is consumed upon the activity of several enzymes including sirtuins (SIRTs), poly-ADP-ribose polymerases (PARPs) and cyclic ADP-ribose synthases (cADPRSs) (Houtkooper* et al*. 2010, Dolle* et al*. 2013). This new found purpose highlighted that NAD^+^, previously thought of as a stable molecule, is actually in continuous turnover and tightly regulated to maintain metabolic homeostasis. In fact, with approximately 2.5 ATP molecules formed per pair of electrons donated by NADH in the electron transport chain, and an estimated 80 kg of ATP turned over by an adult male each day, it is predicted as much as 30 kg NADH is generated every day, reinforcing the need for regulated maintenance of NAD(H) biosynthetic pathways (Kornberg 1991, Walsh* et al*. 2018).

SIRTs are important for metabolic energy control, whereby induction of SIRTs results in posttranslational modifications, such as deacetylation of substrate lysine residues, typically in an NAD^+^-dependent manner (Dali‐Youcef* et al*. 2007, Houtkooper* et al*. 2012). Following activation, generally at times of energy deficit and concomitant with a rise in NAD^+^ content, SIRT activity (most notably SIRT1 and SIRT3) leads to metabolic adaptation in favour of more efficient ATP generation for example by enhancing mitochondrial content and capacity (Rodgers* et al*. 2005, Rodgers & Puigserver 2007, Feige* et al*. 2008, Canto* et al*. 2009, 2010, Hirschey* et al*. 2010, Jing* et al*. 2011, 2013). In models of SIRT1 and SIRT3 deficiency, metabolic impairments were detected particularly following metabolic challenge (Boily* et al*. 2008, Hirschey *et al*. 2010, Xu* et al*. 2010, Kendrick* et al*. 2011, Purushotham* et al*. 2012, Jing *et al*. 2013). However, muscle and liver-specific models of SIRT3 deficiency did not exhibit a metabolic phenotype despite detectable hyperacetylation of mitochondrial proteins; this was potentially explained by compensation by SIRT3 activity in other tissues or key residues unaffected by hyperacetylation (Fernandez-Marcos* et al*. 2012). PARPs, on the other hand, belong to the ADP-ribosyltransferase family and sequentially transfer ADP-ribose from NAD^+^ to proteins, leading to poly ADP-ribosylation (PARylation) (Decker & Muller 2002, Luo & Kraus 2012). Seventeen different PARPs have been identified in mammals and although they have been recognised to have numerous distinct functions the most well-described function of PARP activity is during DNA repair processes (Decker & Muller 2002). PARP1 is activated by DNA damage and PARylation at these sites leads to recruitment and induction of proteins essential to DNA repair processes (El-Khamisy* et al*. 2003, Mao* et al*. 2011, Luo & Kraus 2012). Although not necessarily NAD^+^ dependent, PARP signalling consumes large quantities of cellular NAD^+^ and exhibits a higher affinity for NAD^+^ than SIRTs therefore prolonged PARP activation may deplete cellular NAD^+^ levels (Charron & Bonner-Weir 1999, Ha & Snyder 1999, Kauppinen* et al*. 2013). Though less well defined, the cADPRSs CD38 and CD157 have also been identified as major NAD^+^-consuming enzymes (Aksoy* et al*. 2006, Graeff* et al*. 2009). They are transmembrane enzymes and play vital roles in several physiological mechanisms including maintaining calcium (Ca^2+^) homeostasis and immune function (De Flora* et al*. 1998, Graeff *et al*. 2009).

## NAD^+^ compartmentalisation

Distinct subcellular NAD(H) pools were first proposed due to organelle-specific expression of different NAD^+^-generating and -consuming enzymes (VanLinden* et al*. 2015, Cambronne* et al*. 2016). Technical advances, most recently in the form and use of fluorescent NAD^+^ biosensors, have provided support for the idea of compartmentalisation of independent NAD^+^ pools within cells. Nucleic and cytosolic NAD^+^ levels are typically found to be interchangeable with NAD^+^ able to pass freely through nuclear membrane pores. Conversely, the mitochondrial membrane is impermeable to NAD^+^ and NADH thus mitochondrial NAD^+^ levels fluctuate independently (Cambronne *et al*. 2016). A mitochondria-specific pool of NAD^+^ would seem to enable cells to defend oxidative phosphorylation capacity against transient cytoplasmic/nuclear NAD^+^ depletion (Pittelli* et al*. 2010). NAD^+^ pool size varies in different tissues, for example, cardiac myocytes have an expectedly higher percentage of mitochondrial NAD^+^ compared to cytosolic yet cell types including astrocytes and hepatocytes have a more substantial cytosolic pool (Alano* et al*. 2007). These differences would seem to reflect the demand on oxidative capacity and function of the different cells. Despite individual NAD^+^ pools, vital crosstalk exists between compartments. Glyceraldehyde-3-phosphate and malate–aspartate shuttles allow the transfer of electrons from cytosolic NADH into the mitochondria and oxidised NAD^+^ is released into the cytosol. Thus, cytosolic NAD^+^ concentrations are an important determinant of mitochondrial NADH flux; meaning major loss of NAD^+^, especially in cells predominantly using carbohydrates as a fuel source, would eventually lead to impaired energy production and ultimately cell death (Easlon* et al*. 2008).

## Established NAD^+^ biosynthesis pathways

**Figure 1:**
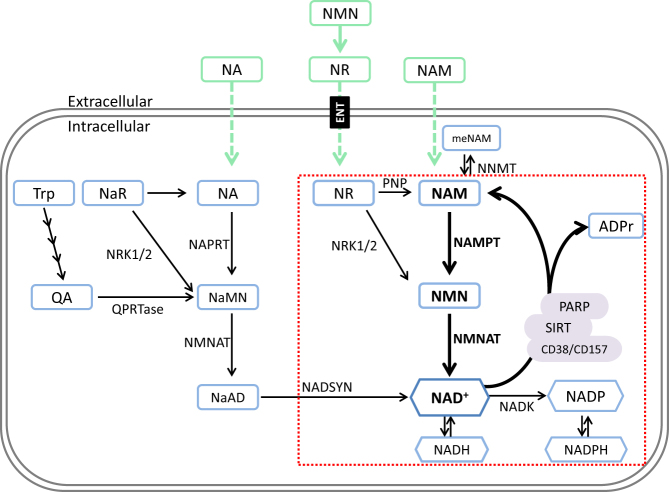
Mammalian NAD^+^ biosynthesis pathways. An illustration of mammalian NAD+ biosynthesis pathways with exogenous precursor supplementation strategies outlined in green with dashed arrows). NAD+ can be generated from Trp *de novo* with multiple enzymatic reactions leading to the production of quinolinic acid (QA), which is then converted to nicotinic acid mononucleotide NaMN by QA-phosphoribosyltransferase (QPRTase) and then to nicotinic acid dinucleotide (NaAD) by nicotinamide mononucleotide adenylyltransferase (NMNAT) activity, before the final conversion to NAD+ by NAD synthase (NADSYN). Alternatively NAD+ can be salvaged from precursors and nicotinic acid (NA) nicotinic acid riboside (NaR) by NA phosphoribosyltransferase (NAPRT) nicotinamide riboside kinases (NRK1/2) respectively to NaMN and finally NDA+ by NMNAT. Alternatively, most tissues rely on the amidated routes for NAD+ biosynthesis (outlined in red box). Here, nicotinamide riboside (NR) and nicotinamide (NAM) are salvaged by NRKs and nicotinamide phosphoribosyltransferase (NAMPT) respectively to nicotinamide mononucleotide (NMN) and ultimately converted to NAD+ by NMNAT.

In mammalian cells, a number of NAD^+^ biosynthesis pathways have been established. These pathways can synthesise NAD^+^ *de novo* from l-tryptophan or from the salvage of vitamin B3 NAD^+^ precursors nicotinic acid (NA), nicotinamide (NAM) and nicotinamide riboside (NR) (Houtkooper *et al*. 2010, Dolle *et al*. 2013) (Fig. 1). The NAD^+^ biosynthesis pathways can be grouped into two distinct routes described as ‘amidated’ or ‘deamidated’ (Mori* et al*. 2014). *De novo* synthesis from tryptophan and NA salvage is grouped into the ‘deamidated’ pathway and shares a final rate-limiting amidation enzyme NADsynthase1 (NADSYN) (Preiss & Handler 1958). Conversely, NAM and NR contain an amide group and therefore follow the ‘amidated’ route to NAD^+^ (Bieganowski & Brenner 2004, Tan* et al*. 2013, Mori *et al*. 2014) (Fig. 1). The high turnover of NAD^+^ in most cell types means that a major disruption to these biosynthesis pathways could lead to a severe depletion of cellular NAD^+^ levels and, if sustained, ultimately lead to cell death (Hasmann & Schemainda 2003, Yang* et al*. 2007).

## *De novo* biosynthesis

*De novo* NAD^+^ biosynthesis predominantly begins with the dietary amino acid l-tryptophan. Initially, N-formylkynurenine is generated from tryptophan by the rate-limiting enzyme indoleamine 2,3-dioxygenase or alternatively tryptophan 2,3-dioxygenase. Next, a sequence of four enzymatic reactions convert N-formylknurenine to α-amino-β-carboxymuconate-ε-semialdehyde (ACMS) (Nikiforov* et al*. 2011). ACMS can either undergo complete oxidation or spontaneous cyclisation to produce quinolinic acid. The conversion of quinolinic acid to nicotinic acid mononucleotide (NAMN) is catalysed by quinolinate phosphoribosyl-transferase and NAMN is subsequently converted to nicotinic acid adenine dinucleotide (NAAD) by the ubiquitously expressed enzyme nicotinamide mononucleotide adenylyltransferase (NMNAT) (Nikiforov *et al*. 2011). Finally, NAAD is amidated to NAD^+^ in an ATP-dependent reaction via glutamine-dependent NADSYN activity (Hara* et al*. 2003).

## Vitamin B3 salvage pathways

Although *de novo* biosynthesis is a major route to NAD^+^ in the liver (Bender* et al*. 1982, Bender & Olufunwa 1988), the majority of cellular NAD^+^ content in other tissues, and to some degree in the liver, is attributed to salvage pathways which resynthesise NAD^+^ from the vitamin B3 precursor molecules NA, NAM and NR, also known as niacins (Mukherjee* et al*. 2017). These precursors share similar chemical structures and can either be absorbed from the diet or found as a consumed NAD(P)(H) metabolites (Gross* et al*. 1998, Bieganowski & Brenner 2004, Khan* et al*. 2006).

NA can be salvaged to NAD^+^ via the Preiss–Handler pathway; initially NA is converted to NAMN in a reaction is catalysed by NA phosphoribosyltransferase (NAPT) with 5-phospho-α-d-ribose-1-diphosphate as its substrate (Preiss & Handler 1958). The pathway then converges with the *de novo* biosynthesis pathway; thus, NAMN is converted to NAAD by NMNAT activity and finally to NAD^+^ by NADSYN (Preiss & Handler 1958). Mammalian NADSYN is a homohexameric protein localised to the cytosol. Its enzymatic activity requires co-substrates ATP and glutamine to convert NAAD to NAD^+^ and releases AMP, PPi and glutamic acid in the process (Mori *et al*. 2014). NADSYN is the final rate-limiting step of both the *de novo* and Preiss–Handler pathway and thus essential for the generation of NAD^+^ via these routes. NADSYN expression and activity has been shown to be both organism and tissue specific. Some microorganisms including *Mycobacterium tuberculosis* are dependent on NADSYN for NAD^+^ biosynthesis, which consequently makes it a potential antibacterial target, whereas others use alternative pathways for NAD^+^ biosynthesis (Boshoff* et al*. 2008, De Ingeniis* et al*. 2012). The rate of NADSYN enzyme activity has been determined in a range of mouse tissues with highest activity levels found in liver and kidney tissue, whereas activity was minimal in brain homogenates and undetectable in lung and skeletal muscle tissues suggesting NAD^+^ biosynthesis via the *de novo* and Preiss–Handler pathways is limited to certain tissues (Mori *et al*. 2014).

Alternatively, NAD^+^ can be salvaged from NAM by the nicotinamide phosphoribosyltransferase (NAMPT) pathway (Tan *et al*. 2013). In this pathway, NAMPT catalyses the first rate-limiting reaction that converts substrates NAM and 5′-phosphoribosyl-1-pyrophosphate (PRPP) to nicotinamide mononucleotide (NMN) in an ATP-dependent reaction. Then, NMNAT activity allows the conversion of NMN to NAD^+^ (Garten* et al*.). Importantly, NAD^+^ consumption by SIRT and PARP activity results in the release of NAM as a reaction product; therefore, salvage by NAMPT provides a central cellular NAD^+^ recycling loop. Recycling of NAD^+^ via this pathway is tightly regulated in order to sustain appropriate NAD^+^ homeostasis, with both NAMPT expression and activity shown to be modulated by cellular NAD^+^ levels and SIRT activity negatively regulated by NAM levels (Bitterman* et al*. 2002).

NAMPT is a dimeric type II phosphoribosyltransferase that is highly conserved and expressed in nearly all the tissues and cells examined suggesting a vital role for NAMPT activity in normal cell function (Wang* et al*. 2006, Garten* et al*. 2015). Initially, it was termed pre-B cell colony-enhancing factor (PBEF) due to its proposed cytokine function (Samal* et al*. 1994); it was then determined to be a hormonal factor, and renamed visfatin, that supposedly exerted insulin mimetic effects (Fukuhara* et al*. 2005) that have since been found to be unverified (Harasim* et al*. 2011). NAMPT is both an intracellular and extracellular protein, the intracellular isoform is predominantly localised to the cytosolic and nucleic fractions (Kitani* et al*. 2003, Revollo* et al*. 2007). Within several cell types NAMPT has been shown as a vital enzyme for maintaining cellular NAD^+^ and energy homeostasis by recycling NAM to NAD^+^ (Yoon* et al*. 2015, Frederick* et al*. 2016, Lin* et al*. 2016, Agerholm* et al*. 2018, Nielsen* et al*. 2018). Pharmacological inhibition of NAMPT using the potent inhibitor FK866 results in NAD^+^ depletion and for cells that predominantly rely on this pathway for NAD^+^ salvage can result in cell death (Hasmann & Schemainda 2003, Tan *et al*. 2013). The importance of extracellular NAMPT (eNAMPT) remains unclear. It has been proposed that extracellular activity may be part of a wider pathological process whereby ATP and PRPP – both required for NAM salvage and normally found at low levels in the plasma – are released from dying cells into the circulation and drive enzyme activity (Hara* et al*. 2011, Garten *et al*. 2015). In diabetes, an association between eNAMPT and pro-inflammatory responses including the induction of inducible nitric oxide synthase has been reported (Kieswich* et al*. 2016).

## The emergence of the NRK pathway to NAD^+^

In healthy cells, the vitamin B3 NAD^+^ precursors are essential for replenishing NAD^+^ for the maintenance of appropriate cellular NAD^+^ levels in all compartments supporting energy homeostasis (Bender & Olufunwa 1988, Bogan & Brenner 2008, Dolle *et al*. 2013). The significance of a requirement for vitamin B3 was first delineated in the disease pellagra, a disorder of B3 deficiency, and since then, increasing evidence has shown that a breakdown in the balance of NAD^+^ generation vs NAD^+^ consumption is a common factor in numerous morbidities (Pitche 2005).

**Figure 2:**
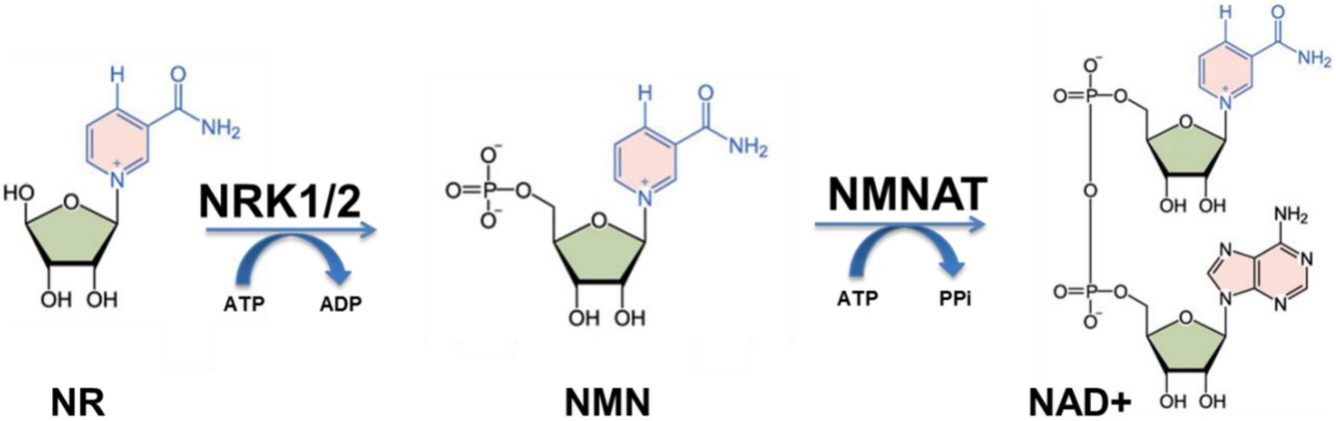
NRK1/2 mediated NAD^+^ biosynthesis pathway. Nicotinamide riboside (NR) is metabolised by nicotinamide riboside kinase (NRK1/2) to nicotinamide mononucleotide (NMN) and subsequently converted to NAD^+^ by NMN-adenylyltransferase (NMNAT) activity.

In 2004, a new pathway to NAD^+^ synthesis was determined by Charles Brenner following the identification of nicotinamide riboside kinase 1 (Nrk1) in yeast and subsequent human homologs NRK1 and NRK2 (Bieganowski & Brenner 2004, Tempel* et al*. 2007). In the initial step of the pathway, NRK activity catalyses the phosphorylation of NR to nicotinamide mononucleotide (NMN) (Tempel *et al*. 2007), which is subsequently converted to NAD^+^ by NMNAT (Fig. 2) (Bieganowski & Brenner 2004). Alternatively, NRK activity can phosphorylate nicotinic acid riboside (NaR) to nicotinic acid mononucleotide (NaMN), which is then converted to NaAD and finally NAD^+^ by NMNAT and NADSYN, respectively (Tempel *et al*. 2007). The discovery of the NRK enzymes stemmed from previous work that showed NR, as a novel NAD^+^ precursor, could extend the lifespan of yeast through the induction of Sir2 in an NAD^+^-dependent manner (Bieganowski & Brenner 2004). Since then, the mounting evidence towards the beneficial effects of using NAD^+^ precursors, and in particular, NR and NMN, has led to the emergence of the NRK enzymes as therapeutic targets. Although the NRK enzymes were identified as an alternative salvage pathway to NAD^+^ (Bieganowski & Brenner 2004), investigations have only recently begun to define the importance of NRK1 and 2 in terms of NAD^+^ metabolism and beyond.

NRK1 and 2 (encoded by *Nmrk1* and *Nmrk2* genes, respectively) both exhibit a high affinity for NR, yet, their substrate specificity differs (Fig. 1). With ATP as a co-substrate NRK1 has a *K*_M_ of 0.088 mM for NR, whereas NRK2 has a *K*_M_ of 0.19 mM (Tempel *et al*. 2007). Furthermore, NRK1 can also use GTP (guanosine triphosphate) as a co-substrate for the phosphorylation of NR yet NRK2 is restricted to ATP (Tempel *et al*. 2007, Dölle & Ziegler 2009). The enzyme turnover of NR to NMN with ATP has also been estimated; here, the *K*_cat_ was found to be around 0.6/S for NRK1 and 0.75/S for NRK2 (Tempel *et al*. 2007). On the other hand, NRK1 and NRK2 exhibit a similar affinity for NaR with a *K*_M_ of 0.051 mM and 0.063 mM, respectively (Fig. 1 and Table 1). Further kinetic studies using a pyruvate kinase/lactate dehydrogenase-coupled enzyme assay confirmed these findings with the *K*_M_ of NRK1 and 2 found to be in the micromolar range and NRK1 again exhibiting a higher affinity for NR. Interestingly, here they also showed that NRK2 had a 10-fold higher affinity than NRK1 for uridine; yet, they share a similar affinity for cytidine (Dölle & Ziegler 2009).

**Table 1:** Estimated NRK1 and NRK2 enzyme kinetics and substrate specificity (Tempel* et al*. 2007).

Substrate	*K*_M_ (mM)	*K*_cat_ (/S)	*K*_cat_/*K*_M_ (/S/M)	Substrate specificity
NRK1				
NR + ATP	0.088	0.6	6800	ATP, GTP
NR + GTP	0.068	0.34	5000	
NaR + ATP	0.051	0.21	4100	
NRK2				
NR + ATP	0.19	0.75	3900	ATP
NR + GTP	30	1.7	57	
NaR + ATP	0.063	0.34	5400	

In mammalian tissues, NRK1 is ubiquitously expressed, whereas NRK2 has been shown to be muscle specific with expression more predominant in skeletal muscle compared to cardiac (Fletcher* et al*., Ratajczak* et al*. 2016). Initial studies in zebrafish identified that Nrk2b is a vital component for musculoskeletal development. In Nrk2b-deficient zebrafish muscular abnormalities were identified, including irregular laminin polymerisation at myotendinous junctions and abnormally long muscle fibres that have elongated into adjacent myotomes (Goody* et al*. 2010). These changes were caused by aberrant cell matrix adhesion complex signalling driven by atypical localisation of Paxillin. It is proposed that the Nrk2b homolog is specifically localised to myotendinous junctions to provide a local NAD^+^ supply, and this may potentially explain why the alternative Nrk homologs are unable to compensate. Importantly, this phenotype was rescued following administration of NAD^+^ demonstrating that the effects were NAD^+^ dependent and that Nrk2b plays a central role in zebrafish NAD^+^ biosynthesis (Goody* et al*. 2012).

However, murine NRK1 and NRK2 loss-of-function models do not exhibit any gross phenotypic abnormalities, with steady state NAD^+^ levels unaffected, at least in the tissues that have been examined (liver, skeletal muscle, brown adipose and kidney) (Fletcher *et al*., Ratajczak *et al*. 2016). Thus, despite the NRK salvage pathway being highly conserved (Bieganowski & Brenner 2004), it appears to play a redundant role in basal murine metabolism; with most tissues examined reliant on the NAMPT salvage pathway to maintain NAD^+^ levels (Fletcher *et al*., Tan* et al*. 2015, Frederick *et al*. 2016, Stromsdorfer* et al*. 2016, Zhang* et al*. 2017, Agerholm *et al*. 2018, Nielsen* et al*. 2018). The differences seen between zebrafish and murine models following loss of NRK function may be attributed to an absence of alternative NAD^+^ biosynthesis pathways in zebrafish skeletal muscle tissue. Although NRK1 and NRK2 do not appear critical in mice for endogenous NR salvage to NAD^+^, their activity has been determined essential for the utilisation of exogenous NR and, more surprisingly, NMN. Following supplementation, it is thought NR is transported into cells via the nicotinamide riboside transporter orthologue of the yeast Nrt1 protein (Belenky* et al*. 2011).

Without expression of the NRK enzymes in tissues, the NAD^+^-boosting effects of NR and NMN supplementation is blocked, whilst expression of alternative NAD^+^ biosynthesis enzymes remains comparable to WT mice (Fletcher *et al*., Ratajczak *et al*. 2016). In addition, the NAD^+^ boosting effect of NR and NMN supplementation following NAMPT inhibition by FK866 in muscle and hepatic cells is comparable to that of untreated cells. Interestingly, phosphorylation of NR by NRK1 appears preferred to NRK2 even in skeletal muscle where *Nmrk2* is specifically expressed and found at substantially higher mRNA levels than *Nmrk1* (Fletcher *et al*.). This firstly shows the NRK enzymes exclusively metabolise NR, but it also suggests that NMN must be converted to NR to enter the cell and then be re-phosphorylated intracellularly back to NMN (Fletcher *et al*., Ratajczak *et al*. 2016). Previous studies have shown that NMN can be dephosphorylated by cytosolic 5′-nucleotidases and thus it has been postulated that NMN is dephosphorylated to NR extracellularly by the cell membrane protein CD73 (also known as ecto-5-nucleotidase) and then imported into the cell (Kulikova* et al*. 2015).

## Organism and tissue NAD^+^ salvage pathway specificity

NAD^+^ biosynthesis and salvage enzymes are evolutionarily conserved from bacteria to mammals yet in some organisms certain pathways are preferred (Berger *et al*. 2004). For example, in mammals, the NAMPT-mediated NAD^+^ biosynthesis pathway is essential for maintaining adequate cellular NAD^+^ levels and complete absence of the *Nampt* gene is embryonically lethal (Garten *et al*. 2015, Tan *et al*. 2015, Zhang *et al*. 2017). Despite being well conserved in mammals, the NRK enzymes appear non-essential yet some microorganisms rely on NRK activity to salvage NAD^+^ (Fletcher *et al*., Gazzaniga* et al*. 2009, Ratajczak *et al*. 2016). For example, the multifunctional protein NadR is expressed in microorganisms and exhibits both ribosylnicotinamide kinase (RNK) activity to phosphorylate NR or NaR, as well as Nmnat activity for NAD^+^ biosynthesis. In *Haemophilus influenzae*, NadR activity is essential for growth yet in microorganisms such as *Salmonella enterica* serovar Typhimurium, NadR activity is present but not vital (Kurnasov* et al*. 2002).

The relative importance and tissue specificity of the different precursor molecules in NAD^+^ replenishment *in vivo* is becoming well defined in some tissues but is unclear in others. For example, *de novo* biosynthesis is thought to be the preferred route for NAD^+^ biosynthesis in the liver (Bender *et al*. 1982, Bender & Olufunwa 1988), with hepatocyte NAD^+^ content potentially more resistant to loss of NAMPT function compared to tissues such as muscle and fat (Schuster* et al*. 2015, Ratajczak *et al*. 2016). With that said, changes to NAMPT expression in hepatocytes has been shown to alter NAD+ content and induce physiological changes highlighting that the NAMPT salvage pathway still plays an important role in hepatocyte NAD^+^ biosynthesis (Zhou* et al*. 2016, Mukherjee *et al*. 2017, Zhang* et al*. 2017). In the majority of other tissues investigated to date, it is thought that cellular NAD^+^ is predominantly attributed to NAD^+^ salvage pathways using vitamin B3 precursors found in the diet or as consumed NAD^+^ metabolites (Mori *et al*. 2014). In many of these tissues, direct recycling of NAM is believed to be preferred over NA salvage; with only transient increases in NAD^+^ following intraperitoneal injection of NA compared to stable increases following NAM administration (Collins & Chaykin 1972). This is further supported by evidence showing NADSYN activity, the final rate-limiting enzyme in the NA salvage pathway appears inferior to the final rate-limiting step of the amidated pathways (Mori *et al*. 2014). In mammalian tissue, NR salvage to NAD^+^ via the NRK enzymes is not essential and appears limited to NR availability with endogenous NR levels seemingly low in tissues yet exogenous delivery of NR appears highly effective in enhancing NAD^+^ directly through NRK activity (Fletcher *et al*., Ratajczak *et al*. 2016).

## Regulation of the NRK enzymes

**Figure 3:**
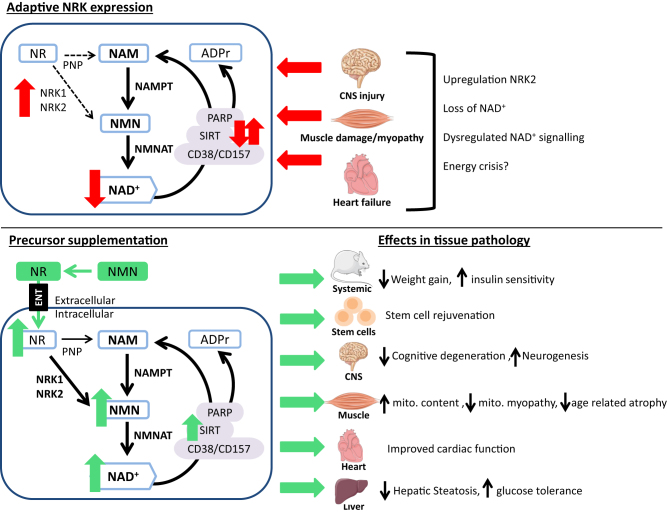
Proposed NRK expression in disease and potential therapeutic interventions. Adaptive NRK expression (Top) has been identified in numerous pathological scenarios where commonly an induction of NRK2 expression has been seen with loss of NAD+ and altered activity of NAD+-consuming enzymes. Supplementation with NAD+ precursors nicotinamide riboside (NR) and nicotinamide mononucleotide (NMN) (bottom), both requiring NRK activity, can elevate intracellular NAD+ and has been shown to result in many health benefits in numerous rodent models.

Aside from the requirement for NRK1 and/or NRK2 to enhance cellular NAD^+^ content following exogenous NR or NMN supplementation, recent data suggest NRK2 may play a an important endogenous role in adaptation to metabolic and energy stress. The regulation of NRK2 has been determined in pathophysiological settings and in response to NAD^+^ insufficiency (Fig. 3 – upper panel) (Fletcher *et al*., Sasaki* et al*. 2006, Lavery* et al*. 2008, Aguilar* et al*. 2015, Xu* et al*. 2015, Diguet* et al*. 2017). Following injury to dorsal root ganglion neurons, *Nmrk2* was the most upregulated NAD^+^ biosynthetic gene (by over 20-fold), which is surprising considering *Nmrk2* expression is typically absent or detected at low levels in central nervous system tissues (Sasaki *et al*. 2006). In addition, in a mouse model of traumatic lower limb muscle injury, *Nmrk2* was again found to be significantly upregulated (three-fold) at 24 h post injury (Aguilar *et al*. 2015). Mice with a loss of hexose-6-phosphate dehydrogenase (H6PDH) function, an ER-based enzyme required for local NADPH generation, manifest with severe muscle myopathy and on transcriptional analysis *Nmrk2* expression was the most dysregulated gene (upregulated by >60-fold mRNA level) (Lavery *et al*. 2008). Furthermore, *Nmrk2* mRNA expression was substantially induced (>80-fold) in models of lethal cardiomyopathy (Xu *et al*. 2015). In this model, administration with NR was able to increase lifespan of these mice by 50% suggesting that this induction of *Nmrk2* in cardiac tissue is to support NAD^+^ generation (Xu *et al*. 2015). The hypothesis of *Nmrk2* induction to support NAD^+^ biosynthesis during injury or potentially extreme energetic stress is further supported by its induction during NAD^+^ depletion. For example, loss of NAD^+^ by pharmacological inhibition of NAMPT also leads to an induction in *Nmrk2* expression, which is normalised when NAD^+^ levels are restored with NR supplementation (Fletcher *et al*.).

More recently, in two murine models of the failing heart that cause dilated cardiomyopathy or cardiac hypertrophy, a significant induction of NRK2 – by 40-fold and 4-fold respectively – was determined (Diguet *et al*. 2017). Conversely, NAMPT expression was downregulated and NAD^+^ levels were reduced in both models. Similar changes were seen in human failing hearts (Diguet *et al*. 2017). These findings led to the proposal by Diguet *et al*. that in NAD^+^ deficiency, NRK2 may be induced to aid NAD^+^ biosynthesis. Circulating NR levels are thought to be low or absent (Trammell* et al*. 2016*a*) but importantly upon dietary NR supplementation protective effects to cardiac function were seen (Diguet *et al*. 2017). They went on to describe the first mechanisms regulating mammalian NRK2 expression. Upregulation of *Nmrk2*, in response to ATP and NAD^+^ depletion, suggested *Nmrk2* is controlled by energy metabolism signalling pathways (Diguet *et al*. 2017). Supporting this hypothesis, they found that *Nmrk2* was regulated during energetic stress in an AMP-kinase and peroxisome proliferator-activated receptor α (PPARα)-dependent manner in cardiac cells (Diguet *et al*. 2017).

Intracellular NAD^+^ concentrations, as well as *Sirt1* and *Nampt* gene expression, have been shown in the liver to be regulated by the circadian clock oscillating in a 24-h rhythm (Nakahata* et al*. 2009). The expression of these genes and NAD^+^ levels are impacted by circadian clock gene loss of function and in reverse pharmacological inhibition of NAMPT and loss of NAD^+^ impacted on circadian gene expression profiles (Nakahata *et al*. 2009). In addition high-fat diet-fed mouse livers saw remodelling of circadian clock expression and ablation of circadian NAD^+^, further linking the importance of NAD^+^ regulation for appropriate circadian clock functioning (Nakahata *et al*. 2009, Eckel-Mahan* et al*. 2013). Less is known about the circadian regulation of *Nmrk1* and 2. *Nmrk1* was recently shown to be circadian regulated in mouse liver tissue and interestingly, like *Nampt*, *Nmrk1* expression was dysregulated in clock-disrupted mice (Mauvoisin* et al*. 2017).

## NAD^+^ precursors as therapeutic interventions

NAD^+^ has long been associated with disease with symptoms of pellagra first described in the 1800s (Pitche 2005). Pellagra is most commonly caused by malnutrition – more specifically a deficiency in vitamin B3 NAD^+^ precursors – and is classically characterised by the triad of symptoms; dermatitis, diarrhoea and dementia. Pellagra is historically associated with diets rich in maize, which presents little nutritional value and it was later found that dietary supplementation with NAD^+^ precursors successfully treated pellagra (Pitche 2005, Naveen* et al*. 2013).

Since the role of NAD^+^ as a central metabolic signalling molecule was discovered, there has been a substantial number of studies associating NAD^+^ availability to metabolic health. A decline in NAD^+^ in multiple tissues and cell types has been linked to the pathophysiology of ageing and chronic disease states including type II diabetes, neurodegeneration, liver disease and cachexia (Koltai* et al*. 2010, Braidy* et al*. 2011, Yoshino* et al*. 2011, Massudi* et al*. 2012, Gomes* et al*. 2013, Long* et al*. 2015, Camacho-Pereira* et al*. 2016, Mills* et al*. 2016, Zhou *et al*. 2016). Many of these studies implicate reduced SIRT expression and/or activity following a loss of NAD^+^ and consequently the activity of key SIRT-mediated targets (Koltai *et al*. 2010, Braidy *et al*. 2011, Yoshino *et al*. 2011, Massudi *et al*. 2012, Gomes *et al*. 2013, Long *et al*. 2015, Camacho-Pereira *et al*. 2016, Zhou *et al*. 2016). Loss of NAD^+^ in many of these scenarios may, at least in part, be attributed to elevated PARP activity (Pillai* et al*. 2005, Braidy *et al*. 2011, Massudi *et al*. 2012, Lehmann* et al*. 2016). With increased DNA damage, a hallmark factor of ageing and a consequence of chronic disease, increased PARP activity to induce DNA repair processes can be detrimental to metabolic homeostasis with a decline to cellular NAD^+^ availability leading to reduced SIRT activity (Decker & Muller 2002, Bai* et al*. 2011*a*, Massudi *et al*. 2012). The importance of NAD^+^-consuming enzyme crosstalk has previously been established through models of PARP excess and deletion. These models demonstrate a concomitant decrease in NAD^+^ and SIRT activity following PARP overexpression and an increase in NAD^+^ content and SIRT-mediated signalling when PARP activity is inhibited (Mangerich* et al*. 2010, Bai *et al*. 2011*a*,*b*, Luo & Kraus 2012, Mohamed* et al*. 2014). PARP inhibitors have already shown promise as anticancer agents and now may also be valuable therapeutics against metabolic disease, with PARP inhibition resulting in enhanced oxidative metabolism in mouse models (Bai *et al*. 2011*a*,*b*, To* et al*. 2014). Recent studies have also implicated CD38 in age-related mitochondrial dysfunction. An increase in CD38 expression was determined in metabolic tissues such as liver, adipose and muscle. Detrimental effects of CD38 in ageing, mediated via a SIRT3 mechanism, were to some extent reversed in CD38 loss-of-function mouse models (Camacho-Pereira* et al*. 2016).

The knowledge that enhancing NAD^+^ can induce positive metabolic effects mediated through the induction of SIRT has additionally resulted in a vast array of studies looking at the efficacy of using NAD^+^ precursors to boost NAD^+^ (Mouchiroud* et al*. 2013, Fang* et al*. 2017, Rajman* et al*. 2018). NA derivatives have been used clinically for many years, notably in treatment of hypercholesterolemia; however, action on the GPR109A receptor causes undesirable flushing side effects in a large proportion of patients and ultimately leads to poor compliance (Taskinen & Nikkila 1988, Gille* et al*. 2008, van de Weijer* et al*. 2015). There has since been major interest in the use of alternative NAD^+^ precursors that do not activate the GPR109A. Although nicotinamide has been shown to be effective in boosting NAD^+^ in tissues, concerns regarding the requirement of high doses, which may exert an inhibitory effect on SIRT activity and reduce efficacy (Collins & Chaykin 1972, Bender & Olufunwa 1988, Bitterman *et al*. 2002, Liu* et al*. 2009, Mitchell* et al*. 2018), have led to a surge of research focussing on the therapeutic potential of NR and NMN (Elhassan* et al*. 2017).

Both NR and NMN have been shown as effective NAD^+^ enhancers in numerous models following supplementation without any notable adverse effects reported to date (Yoshino *et al*. 2011, Cantó* et al*. 2012, Khan* et al*. 2014, Mills *et al*. 2016, Trammell* et al*. 2016*b*, Uddin* et al*. 2016, Wang* et al*. 2016, Zhang* et al*. 2016). The number of studies investigating their therapeutic potential has grown rapidly in recent years. Although these studies have often focussed on one or the other compound, similar and overlapping effects of either NR or NMN supplementation have been reported (Fig. 3 – lower panel). These include NR-mediated NAD^+^ repletion in ageing mice protecting against muscle degeneration, and similarly delayed muscle stem cell senescence in models of muscular dystrophy through improved mitochondrial function resulting in enhanced regenerative capacity and more favourable phenotypes (Zhang *et al*. 2016). Importantly, the beneficial regenerative effects of NR extended to neural and melanocyte stem cells where it was shown to delay senescence and extend life span (Zhang *et al*. 2016). NMN supplementation also exhibited positive effects on neurogenesis in aged mice by preserving the neural stem/progenitor cell pool (Stein & Imai 2014). In models of cardiac pathology including ischaemia and reperfusion, heart failure and hypertrophy supplementation with either NMN or NR has resulted in improved outcomes (Yamamoto* et al*. 2014, Diguet *et al*. 2017). In metabolic diseases of obesity, diabetes and non-alcoholic fatty liver disease (NAFLD) protective effects of exogenous NR or NMN delivery include enhanced metabolic flexibility, mitochondrial function, improved glucose tolerance, reduced weight gain and prevention of hepatic steatosis and other pathological factors of NAFLD (Yoshino *et al*. 2011, Cantó *et al*. 2012, Trammell* et al*. 2016*b*, Zhou *et al*. 2016, Shi* et al*. 2017) (Fig. 3). There have been many more studies recently published investigating the *in vivo* effects of NR and NMN supplementation in a variety of disease models, and a more detailed analysis of these has been well described in a number of recent reviews (Fang *et al*., Rajman* et al*. 2018).

Currently, the majority of work looking at the benefits of vitamin B3 supplementation has been carried out using animal models; thus, the translational potential to humans is still unclear. Currently, a number of clinical trials focussing on NR and NMN supplementation have recently been completed or are still ongoing. The first clinical study with NR – a double-blind, randomised pharmacokinetic study with 12 participants – found a single oral dose of 1000 mg NR raised circulatory NAD^+^ levels by 2.7-fold; interestingly, NAD^+^ metabolomics also identified NAAD as a highly sensitive biomarker for NAD^+^ (Trammell* et al*. 2016*a*). In another non-randomised clinical study with eight subjects, a dose-dependent increase in NAD^+^ was found with 250–1000 mg/day NR was orally administered over 9 days with a maximal two-fold increase seen in NAD^+^ (Airhart* et al*. 2017). An 8-week double-blind, randomised clinical trial (*n* = 118) assessing the efficacy and safety of repeat dose NR in combination with the polyphenol molecule pterostilbene, collectively known as NRPT, dosed at either 250 mg NR/50 mg PT or 500 mg NR/100 mg PT vs a placebo control group has been conducted (Dellinger* et al*. 2017). This study provided positive results regarding the safety of repeat dose NRPT and also showed a significant increase in whole blood NAD^+^. In addition, potentially positive effects were observed regarding liver function, which may become more apparent in larger scale clinical trials (Dellinger *et al*. 2017). In addition, an in-depth analysis of chronic NR supplementation (500 mg, twice/day) has recently been investigated in a randomised 6-week, double-blind, placebo-controlled, crossover clinical trial in healthy middle-aged to older adults (*n* = 24). The results from this latest trial further supported previous studies, showing that at this dose, chronic NR was well tolerated by subjects and was also able to enhance NAD^+^ levels of blood cells (Martens* et al*. 2018). Interestingly, the study also demonstrated some positive trends following NR supplementation in terms of cardiovascular health including NR potentially reducing systolic blood pressure and arterial stiffness (de Picciotto* et al*. 2016). Again, larger scale clinical trials are now required to establish the potential of NR to exert beneficial effects on these cardiovascular parameters and beyond. There are several ongoing clinical studies that are currently further assessing the safety, efficacy and impact of either NR or NMN supplementation on a wide range of health parameters including vascular endothelial function, immune function, kidney function, muscle mitochondrial function, cognitive function and energy metabolism (see clinicaltrials.gov). Although there is still much to learn regarding the clinical impact of NR and NMN supplementation, currently no adverse effects have been reported (Trammell* et al*. 2016*a*, Airhart *et al*. 2017).

Despite many studies reporting beneficial outcomes in disease models, and the absence of adverse effects from the clinical studies to date, there is still some concern about possible unwanted effects of elevating NAD^+^ with particular focus surrounding immune responses. Numerous mechanistic studies have provided rationale for these concerns with an association found between raised NAD^+^ levels and pro-inflammatory effects (Van Gool* et al*. 2009, Preyat* et al*. 2016, Gerner* et al*. 2017). Notably, secretion of the pro-inflammatory cytokine TNF-α is responsive to intracellular NAD+ levels via a proposed SIRT6-based mechanism with loss of SIRT function able to reverse these effects (Van Gool *et al*. 2009). Although typically thought to be a pro-survival factor, NAD^+^ has also been found to regulate TNF-induced necroptosis, a regulated form of necrosis, via a SIRT-dependent mechanism (Preyat *et al*. 2016). SIRT1 has also been identified to induce pro-inflammatory effects by promoting T helper 17 effector cell generation and function (Lim* et al*. 2015). The use of NAMPT inhibitors to deplete cellular NAD^+^ levels has been explored in models of malignancy and inflammation with some studies showing promising effects (Montecucco* et al*. 2013*b*). For example, in disease models of colitis where NAMPT is upregulated in response to an increased turnover of NAD^+^ as a result of enhanced activity of NAD^+^-consuming enzymes, NAMPT inhibition was able to markedly supress key inflammatory processes (Gerner *et al*. 2017). Similar anti-inflammatory effects have been seen in models of arthritis, experimental autoimmune encephalomyelitis and myocardial ischaemia/reperfusion (Busso* et al*. 2008, Bruzzone* et al*. 2009, Montecucco* et al*. 2013*a*,*b*). Although to date there have been no reports of pro-inflammatory effects following vitamin B3 supplementation, these effects may become apparent following long-term precursor supplementation or in certain inflammatory disease states. As many of these pro-inflammatory mechanisms are thought to be mediated by SIRT activity perhaps the rise in nicotinamide, a known inhibitor of SIRT activity, consistently found following NR and NMN supplementation may provide a means of protection against deleterious effects (Bitterman *et al*. 2002).

## Future research avenues

Understanding of the importance of NAD^+^ in metabolic health has greatly advanced in recent years and currently research is now focussed on further defining firstly the key pathways vital to maintaining NAD^+^ homeostasis but secondly establishing the most useful pathways to manipulate for enhanced NAD^+^ turnover. Many research challenges still remain including organism, tissue and organelle specificity as well circadian signalling and technical limitations for quantification. The identification of more robust biomarkers to monitor NAD^+^ turnover in health and disease would be extremely valuable.

In terms of clinical importance, many questions are still unanswered regarding the translational likelihood of NR and NMN salvage via the NRK enzymes. These include delivery of appropriate levels of substrate to target tissue, the best route of substrate delivery, dosing and timing taking into account circadian fluctuations in enzymes expression.

Furthermore, the function of NRK2 is still unclear, although it appears to play a redundant role in NAD^+^ biosynthesis along with NRK1, at least in unchallenged models, its highly regulated expression particularly in times of stress suggest it may have role beyond NAD^+^ metabolism.

## Concluding remarks

The requirement of NRK1 and 2 to utilise exogenous NR and NMN has led to a new found interest into the regulation of this alternative NAD^+^ pathway. The notion of using NAD^+^ precursors to boost NAD^+^ is now beginning to see translation into human studies, appearing to be safe, naturally available, and having the potential to improve health outcomes across a large range of pathophysiological scenarios. NR and NMN supplementation are at the forefront of several clinical trials as a result of mounting evidence from a vast array of *in vivo* studies supporting their therapeutic potential. Despite this, there is still much more to learn about the NRK enzymes and how best to target NAD^+^ boosting therapies in order for them to maximise their effects. In terms of NAD^+^ biosynthesis, NRK1 appears to play a more crucial role upon exogenous delivery of NR. NRK2 is much more highly regulated with expression typically muscle specific except in times of injury of energetic stress where large changes to expression have been observed. Further work is now necessary to determine whether this induction is a purely adaptive response to acute loss of energy homeostasis or if there is a potential alternative function for NRK2 beyond NAD^+^ metabolism. The induction of NRK activity may also point to the tissues and scenarios in which NR and NMN may be most therapeutically effective.

## Declaration of interest

The authors declare that there is no conflict of interest that could be perceived as prejudicing the impartiality of this review.

## Funding

GGL is supported by a Wellcome Trust Senior Fellowship (grant number-104612/Z/14/Z).
